# Prevalence of Mastitis, Bacteriological Identification, and Antibiogram of *Staphylococcus aureus* From Subclinically Infected Lactating Cows in Tocha and Essera Districts, Dawuro Zone, Southwest Ethiopia

**DOI:** 10.1155/vmi/3884904

**Published:** 2026-10-15

**Authors:** Ephrem Gebrehiwot, Mekonnen Addis, Mukarim Abdurahman, Tamrat Haile, Tsegaye Teklemariam

**Affiliations:** ^1^ Department of Veterinary Medicine Bonga University College of Agriculture and Natural Resource Bonga Ethiopia; ^2^ Jimma University College of Agriculture and Veterinary Medicine Jimma Ethiopia; ^3^ Mizan Regional Veterinary Laboratory Center Mizan Ethiopia

**Keywords:** antimicrobial resistance, Dawuro, Ethiopia, raw milk, *S. aureus*, subclinical mastitis

## Abstract

*Staphylococcus aureus* is a common cause of subclinical mastitis, which has got drug resistance and poses a major threat to public and animals. Inappropriate use of drugs, consumption of raw milk, and lack of updated and published scientific work were out of major problems. A cross‐sectional study was conducted to assess the occurrence of mastitis, and isolate, identify, and assess antimicrobial susceptibility pattern of *S. aureus* from subclinically infected lactating cows in Tocha and Essera districts of Dawuro Zone from January–November 2024. Questionnaire survey, clinical examination, California mastitis test, bacteriological identification, and antimicrobial susceptibility testing were conducted. About 384 cows were selected using a simple random sampling method. The overall prevalence of mastitis was 33.60% (129/384), of which 5.73% (22/384) and 27.86% (107/384) were clinical and subclinical mastitis, respectively. Breed (*p* = 0.007, AOR = 1.985), lactation period (*p* ≤ 0.001, AOR = 3.021, and *p* = 0.030, AOR = 1.872), and previous history of mastitis (*p* = 0.018, AOR = 1.804) significantly associated with subclinical mastitis. The overall prevalence of *S. aureus* in subclinical mastitis was 35.5% (38/107). About 92.1%, 89.47%, 86.84%, 81.57%, and 78.95% of *S. aureus* isolates were susceptible to cotrimoxazole (trimethoprim + sulfamethoxazole), kanamycin, streptomycin, sulfisoxazole, and erythromycin, respectively. However, the isolates were highly resistant to penicillin G 100% (38/38) followed by ampicillin 97.37% (37/38), oxacillin 89.47% (34/38), tetracycline 68.42% (26/38), and oxytetracycline 52.63% (20/38). About 5.26% (2/38) (2/38) were multidrug resistant. The high prevalence of subclinical mastitis with high level of drug‐resistant *S. aureus* in raw milk in this study seems dire, a serious challenge for a resilient tomorrow, and is a threat to us all. Thus, regular screening for subclinical mastitis, testing antimicrobials for susceptibility prior to use, and creating awareness to the community to avoid raw milk consumption and the farmers to practice good animal health and management were recommended.

## 1. Introduction

Ethiopia has the largest livestock population of any African country standing 1^st^ in Africa and 5^th^ in the world with a total population of 210,850,000 [1]. Despite the huge numbers of livestock resource and great potential for increased livestock production, productivity and commercialization of livestock and livestock product are low [2]. Among livestock production systems, dairy production is one of the prevalent production systems and cattle are the main source of milk production and they have their own contribution to family nutrition and income generation [3]. Dairy production has not been fully exploited, mainly due to several constraints including malnutrition, traditional management, and disease such as mastitis [4, 5]

In Ethiopia, the available information indicates that bovine mastitis is one of the most frequently encountered diseases of dairy cows, which contributes to the reduction in milk production in dairy herds [6]. It is a disease resulting from the interaction of various factors associated with the host, pathogen(s), environment, and management. It is estimated that about 150 various bacterial species/subspecies can induce mastitis in dairy cattle. Of these bacteria, *Staphylococcus aureus* is the leading pathogen causing the most dangerous mastitis in cattle and the common cause of dairy product loss in most countries. *S. aureus* has emerged as superbug of dairy udder, compromising animal health and economy [7, 8].

Antimicrobial resistance (AMR) represents a major threat to human and animal health with significant global economic and security implications. In addition to its high prevalence, *S. aureus* is well known for its ability to acquire resistance to a variety of antibiotics. It is evolving pathogens in that it readily acquires drug resistance, and multiple drug‐resistant (MDR) isolates are increasing in animal health and human health [9]. The indiscriminate use of antibiotics for the treatment of animal and human diseases, for the feeding of animals, or as growth promoters has led to the development of AMR, thereby rendering the antibiotic treatment ineffective [10].

Recently published studies on bovine mastitis in Ethiopia reveal the prevalence of mastitis ranging from 29% to 73% ([11–13, 13–19]). On the other hand, studies confirmed that *S. aureus* has got its highest level of resistance against many antimicrobial agents [13, 20–22]. Inappropriate use of drugs by animal health workers without in vitro testing for susceptibility and a large number of farmers treating their sick animals themselves or getting the services from para‐veterinarian due to limited veterinary service were also common in many parts of Ethiopia including the current study area. Moreover, the consumption of raw cow’s milk and its derivatives is also common, which is not safe from the consumer health point of view as it may lead to the transmission of various diseases [23] including antibiotic resistance *S. aureus*. Yet, there is no accessible published scientific study in the current study area related to the disease. Therefore, this study was carried out with the objective of assessing the prevalence of bovine mastitis and associated risk factors and isolating and identifying *S. aureus*, and assessing the antimicrobial susceptibility pattern of *S. aureus* from subclinically infected lactating cows in Tocha and Essera districts of Dawuro Zone.

## 2. Materials and Methods

### 2.1. Study Area

This study was conducted in Tocha and Essera districts of Dawuro Zone in the southwestern region of Ethiopia. Dawuro Zone lies between 6°59′–7°35′ north latitude and 36°6′–37°53′ east longitude with an elevation ranging from 1300 to 3500 masl (Figure 1). The annual temperature ranges from 16°C to 29°C and annual rainfall of 900–1620.3 mm. It consists of 11 districts and two town administrations. Tocha district has 15 kebeles (14 rural and 1 urban kebele), whereas Essera district has 29 kebeles (27 rural and 2 urban). The livestock population of the zone comprises 2,969,877 cattle, 4,474,396 sheep, 619,838 goats, 661,625 equines, and 2,845,477 poultry. District’s data are depicted in Table 1 [24].

**FIGURE 1:**
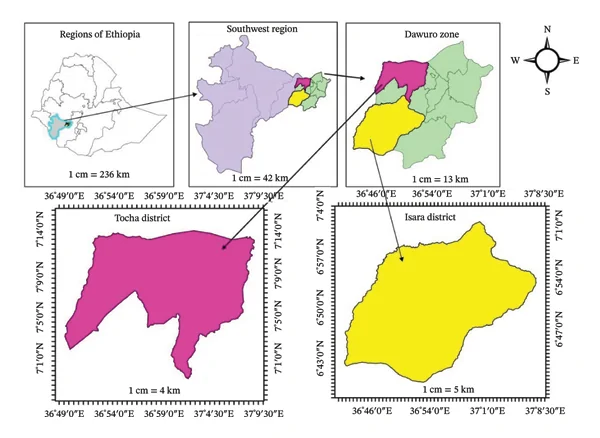
Map of study area (source: prepared by the author using Esri ArcGIS desktop 10.7).

**TABLE 1:** Description of study districts.

	Tocha district	Essera district
Distance from Addis Ababa	521 km	670 km
Location	7.01–7.23N & 36.81–37.15E	6.7–7.06°N & 36.7–37.1°E
Altitude	2400–2870 masl	2020 to 2500 masl.
Cattle population	147,992	550,047

### 2.2. Study Population

The study populations were both indigenous or zebu breed and crossbreed (Holstein Friesian ×  zebu or Jersey × zebu) lactating cows found in the Tocha and Essera District of Dawuro Zone kept under an extensive management system.

### 2.3. Study Design and Period

A cross‐sectional study design was conducted from January to November 2024.

### 2.4. Sample Size Determination and Sampling Procedure

#### 2.4.1. Sample Size Determination

The sample size was determined by using the formula given by Ref. [25]. 95% confidence level and 5% desired absolute precision were used. There was no previous research done in study area; therefore, the expected prevalence of 50% was considered.

(1) Sample size N=Z2×Pex1−Pexd2,

where *n* is the required sample size.

Pexp is the expected prevalence of *S. aureus* occurrence in bovine milk.

*Z* is the *z* statistic for the level of confidence, which is 1.96.

*d* is the absolute error or precision (allowable error 5%).

(2) N=Z2×Pex1−Pexd2 =1.961.960.5×××10.5−0.0052=3.84160.50.5××0.0025 =384.

A total sample size of 384 lactating cows was selected from six selected kebeles of Tocha and Essera district.

#### 2.4.2. Sampling Procedure

Three kebeles from Tocha district, namely, Medhanealem, Edget, and Botori, and three kebeles from Essera district, namely, Bale, Sengeti, and Kambo, were selected purposively on the basis of road accessibility and potential for cattle population. Small holder farmer cattle herds or households in each kebele with lactating cow during the study period were identified and listed before sampling activity. Accordingly, a total of 419 households or herds (61, 86, 74, 69, 58, and 71 herds from Medhanealem, Edget, Botori, Bale, Kambo, and Sengeti kebeles, respectively) were identified during the study period. Lactating cows within these herds/households were selected using a simple random sampling technique. Equal proportion of the samples was taken from six selected kebeles (64 samples from each kebele).

### 2.5. Data Collection

#### 2.5.1. Questionnaire Survey and Observation

A semistructured questionnaire was developed, and the data based on the study objective were recorded. For each sampled cow, data regarding animal identification, farm management, and potential risk factors for the occurrence of subclinical mastitis in dairy cows were collected from farm owners or animal handlers during sample collection. The animal‐level factors such as age, breed, lactation stage, parity, body condition, teat blindness, and presence of teat lesion were also recorded. The farm‐level factors such as herd size, housing types, farm hygiene, previous history of treatment of mastitis, barn floor status, type of milking method, use of towels, milking sequences, and hygiene were recorded. Udder and milk abnormalities (injuries, blindness, tick infestation, swelling, milk clots, abnormal secretion, etc.) were also recorded.

To determine the age of the lactating cows, birth records, information from owner, or dentition characteristics were used and the age of the cow was categorized as young (3 up to 5 years), adult (6 up to 8 years), and old (9 years and above). Parity was categorized as few (1 up to 2 calves), moderate (3 up to 5 calves), and many (6 calves and above) [26–28]). The stage of lactation was categorized as early (1^st^ to 3^rd^ month), mid (4^th^ to 6^th^ month), and late (7^th^ month to the beginning of dry period). Animal’s body condition score was categorized as poor, moderate, and good based on vertebrae at the middle of the back, fat deposit behind the shoulder and in the brisket area, rear view of the hook bone (cross section), side view of the line between hook and pin bones, and cavity between the tail head and pin bone [29].

#### 2.5.2. Diagnosis for Mastitis

##### 2.5.2.1. Clinical Inspection of the Udder and Milk for Clinical Mastitis

Udders of the selected lactating cows were examined by visual inspection and palpation for the presence of any abnormalities. In addition, milk from a cow was checked for any change in color and consistency [30]. Clinical mastitis cases were diagnosed based on the manifestation of signs of inflammation of the udder upon palpation and/or gross changes in milk; and presence of misshaped, atrophied, hard and/or fibrotic udder quarter was diagnosed as chronic mastitis [31].

#### 2.5.3. Screening for Subclinical Mastitis

The California mastitis test (CMT) was used as a screening test for subclinical mastitis. It was conducted according to standard procedures according to the procedures described by the National Mastitis Council [32]. To screen the selected lactating cows, about 2 mL of milk sample from each udder quarters was placed into a plastic paddle separately, and then 2 ml of CMT reagent was added to each and rotated to mix the contents. The CMT result was scored as negative if there was no gel formation and recorded positive if there was gel formation ranging from trace (T), weakly positive (+1), distinct positive (+2), and strongly positive (+3) as per the recommendation given by NMC [33].

### 2.6. Milk Sample Collection, Handling, Storage, and Transportation

The milk sample for the isolation and identification of *S. aureus* was taken from cows identified as positive for subclinical mastitis by CMT. Procedure for the collection of milk was carried out according to Quinn et al. [30]. The milk sample was collected before regular milking. Aseptic procedure was followed during milk samples collection in order to prevent contamination with micro‐organisms. After the hands were cleaned by detergents and clean water, the udder and teats were washed with tap water and disinfected with ethanol (70%) and allowed to dry. During washing and disinfecting, teat on the far side of the udder was cleaned first. Sterile universal bottle with tight fitting cups was used. These universal bottles were labeled with permanent marker before sampling. For bacteriological culture, about 10 mL of milk was collected aseptically from all affected teats together in a universal bottle. The samples were then placed into icebox and transported to the Mizan Regional Veterinary Laboratory where the samples were inoculated to enrichment media or stored at 4°C for a maximum of 24 h until inoculation.

### 2.7. Isolation and Identification of *S. aureus*

#### 2.7.1. Enrichment Medium

Bacteriological examination was carried out according to Quinn et al. [30]. Refrigerated milk samples were allowed to stay at room temperature (25°C) for about an hour and then mixed by shaking in order to disperse bacteria and fat and allowed to stand for a while for foam to disperse before just inoculation. A loopful of milk sample was then streaked on blood agar base enriched with 5% defibrinated sheep blood using the quadrant streaking method and then incubated aerobically at 37°C. The plates were checked for bacterial growth after 24, 48, and 72 h to rule out slow‐growing bacteria. The cultures were examined for growth, morphological features, such as colony size, shape, and color, and hemolytic characteristics.

#### 2.7.2. Gram’s Stain

All suspect cultures of *S. aureus* were subjected to Gram’s stain and observed under a light microscope for Gram’s reaction, size and shape, and cell arrangements. Gram‐positive cocci occurring in bunch, grapelike irregular clusters by Gram’s stain were taken as presumptive *S. aureus*.

#### 2.7.3. Biochemical Tests

##### 2.7.3.1. Catalase Test

In the catalase test, pure culture of the isolates was picked up by loop on a clean slide and mixed with a drop of 3% H_2_O_2_. Liberation of bubbles within a few seconds occurs for positive isolates [30].

##### 2.7.3.2. Selective Plating on Mannitol Salt Agar (MSA) Media

MSA was used as the selective medium for the isolation of *S. aureus*. The samples with grapelike irregular clusters by Gram’s stain and catalase positive were selected and streaked on an MSA plate and incubated at 37°C and examined after 24–48 h for growth. The presence of growth and change in pH in the medium (red to yellow) is regarded as presumptive of *S. aureus*. Fermentation of mannitol by *S. aureus* causes yellow discoloration of the medium within 24 h of incubation [30].

##### 2.7.3.3. Coagulase Test

The presumptively identified *S. aureus* from MSA were subcultured to nutrient agar plate, and after 24 h, colony was picked by the bacteriological loop and placed on a clean slide with a small drop of distilled water and emulsified. The test suspension was also treated with a drop of rabbit plasma and mixed well with a needle for 5–10 s. The colonies forming clumping were taken as coagulase‐positive *S. aureus* [30]. Slide coagulation test–positive samples were subjected to the tube coagulation test for final confirmation as coagulase‐positive *S. aureus* following the procedures.

##### 2.7.3.4. Maltose Fermentation on Purple Agar Base (PAB) Media

PAB supplemented with 1% maltose was used to distinguish types of pathogenic staphylococci, especially coagulase‐positive isolates. Coagulase‐positive Staphylococcus cultures were inoculated on the plate of PAB medium containing 1% maltose and incubated at 37^O^C for 24–48 h and observed for the growth and change of media to yellow. Pathogenic *S. aureus* was identified by its ability to rapidly ferment maltose and produce acid metabolites, which cause the pH indicator (bromocresol purple) to turn the culture medium and colonies to yellow [30].

### 2.8. Antimicrobial Susceptibility Test

Based on the standard procedure of the Clinical and Laboratory Standards Institute [34], the AMR pattern of the *S. aureus* isolates was performed on Mueller–Hinton agar by the Kirby Bauer disk diffusion method. Antimicrobials that were routinely used for the treatment of mastitis in the study areas were considered for susceptibility testing. Drugs were selected from different classes of antimicrobials. Accordingly, about 10 antimicrobials that were commonly used for treatment were selected as indicated in Table 2.

**TABLE 2:** List of antimicrobials selected for the current study.

Class of antibiotics	Name of antimicrobials	Code	Potency
Penicillins (β‐lactams)	Penicillin G	P	10 unit
	Ampicillin	AMP	10 µg
	Oxacillin	OX	1 µg

Aminoglycosides	Kanamycin	K	30 µg
	Streptomycin	S	10 µg

Macrolides	Erythromycin	E	15 µg

Tetracyclines	Oxytetracycline	OT	30 µg
	Tetracycline	TE	30 µg

Sulfonamides	Sulfisoxazole^∗^	SF	300 µg
	Cotrimoxazole (trimethoprim + sulfamethoxazole)	COT	25 µg

^∗^Sulfisoxazole was used to represent any of currently available sulfonamide preparations [35].

About 2–3 pure colonies of *S. aureus* isolates were taken from the nutrient agar plate and transferred to 5 mL of 0.85% saline water. Turbidity was measured using the McFarland densitometer and adjusted to 0.5 McFarland. It was then inoculated by dipping a sterile cotton swab into it and wiped on the Mueller–Hinton agar, according to the standard procedure [34]. The streaking procedure was repeated two more times, and the plate was rotated approximately 60° each time to ensure an even distribution of inoculum. Thereafter, different antimicrobial discs (Table 2) were distributed evenly on the MH plate using a disc dispenser (Oxoid disc dispenser) so that they were not closer than 24 mm from center, and were gently pressed down with the point of a sterile forceps to ensure complete contact with the agar surface. Plates were then incubated at 37°C for 18–24 h. After the final incubation time, the zone of inhibition around each disc was measured with the help of digital caliber, which was held on the back of the inverted petri plate. Finally, the results were interpreted as sensitive, intermediate, and resistant using a standard zone of the interpretative chart [34].

### 2.9. Inclusion and Exclusion Criteria

At the farm level, inclusion criteria targeted actively lactating dairy cows during the study period. Those lactating cows exhibiting either clinical mastitis—characterized by udder swelling, heat, pain, and visible milk changes like clots or flakes or subclinical mastitis, typically defined by a CMT score of 1+ or greater—were included. Conversely, animals were excluded if they are not lactating and had received local or systemic antimicrobial therapy within the preceding 14–30 days, were in the dry or immediate postcalving colostrum phase, suffer from concurrent severe systemic illnesses, or possess blind, nonfunctional quarters. For laboratory and bacterial identification, inclusion criteria required milk samples to be collected under strict aseptic conditions and maintained under a proper cold chain and demonstrate significant monomicrobial or dual‐pathogen growth on primary media. Laboratory exclusion criteria strictly omitted samples showing the growth of three or more distinct bacterial colony types—which signifies environmental or fecal contamination rather than a true intramammary infection—as well as specimens with heavy saprophytic overgrowth, broken cold chains, unreadable labeling, or nonviable subcultures that prevent clear genus‐ or species‐level biochemical identification. Moreover, farmers or cow owners who were willing to participate in the study were included. In contrast, owners who were not interested to cooperate and participate in the study were not included.

### 2.10. Data Management and Analyses

The data generated during the sample collection and from the questionnaire were entered into the Microsoft Excel spreadsheets. Descriptive statistics were used in summarizing quantitative data as appropriate. Logistic regression was used for risk factor association. Prevalence of mastitis, the association among variables were, the association among variables were computed using various statistical tools in IBM SPSS Version 27 software. For all statistical analysis, 95% confidence level and the significant level of *p* value 0.05 were considered.

## 3. Results

### 3.1. Prevalence of Mastitis

From a total of 384 lactating dairy cows examined, 129 (33.60%) were found suffering from mastitis, out of which 22 (5.73%) and 107 (27.86) were found to be infected with clinical mastitis and subclinical mastitis (Table 3).

**TABLE 3:** The prevalence of mastitis in Tocha and Essera districts of Dawuro Zone (January–November 2024).

Type of mastitis	Number positive (%)	Prevalence (*N* = 384) (%)	95% CI
Clinical mastitis	22	5.73	3.60–8.50
Subclinical mastitis	107	27.86	24.90–34.60
Total	129	33.60	28.90–38.60

### 3.2. Factors Associated With Subclinical Mastitis

#### 3.2.1. Animal‐Level Factors

Univariable logistic regression was used to determine the associated cow‐level factors to subclinical mastitis. Among the factors assessed, only breed (*p* = 0.005) and lactation period (*p* ≤ 0.001 for mid‐lactation and *p* = 0.034 for late lactation) showed a trend toward significance for subclinical mastitis (*p* = 0.227), and other factors remained insignificant (Table 4).

**TABLE 4:** Dairy cow‐level factors associated with prevalence of SCM in Tocha and Essera districts of Dawuro Zone (January–November 2024).

Variables	Level/category	Number examined	Number positive	Prevalence	*p* value	COR (95% CI)
Breed	Local	190	44	23.16		Ref.
	Cross breed	172	63	36.63	0.005	1.918 (1.213–3.032)

Age	Young	111	25	22.52	0.146	Ref.
	Adult	150	48	32	0.072	1.746 (0.951–3.204)
	Old	101	34	33.66	0.783	1.078 (0.631–1.844)

Body condition	Good	97	28	28.86	0.981	Ref.
	Medium	197	59	29.95	0.939	1.027 (0.519–2.031)
	Poor	68	20	29.41	0.933	0.975 (0.533–1.783)

Parity	Few (1‐2 calve)	137	33	24.08	0.198	Ref.
	Moderate (3–5 calves)	152	49	32.24	0.118	1.641 (0.881–3.058)
	Many (≥ 6 calves)	73	25	34.25	0.764	1.095 (0.606–1.977)

Lactation period	Early (1–3 months)	127	26	20.47	0.002	Ref.
	Medium (4–6 Months)	124	35	28.22	< 0.001	2.749 (1.55–4.876)
	Late (≥ 7 Months)	111	46	41.44	0.034	1.8 (1.045–3.099)

Udder and leg hygiene	Clean	165	48	29.1	0.981	Ref.
	Slightly dirty	141	42	29.78	0.857	1.062 (0.548–2.058)
	Very dirty	56	17	30.36	0.937	1.027 (0.523–2.017)

Depth of udder	Normal	266	74	27.82		Ref.
	Pendulous	96	33	34.37	0.228	1.358 (0.825–2.239)

Lesion on udder	No	289	83	28.72		Ref.
	Yes	73	24	32.87	0.487	1.216 (0.701–2.109)

Tick on udder	No	217	59	27.2		Ref.
	Yes	145	48	33.1	0.227	1.325 (0.839–2.093)

#### 3.2.2. Farm‐Level Factors Associated With Prevalence of SCM

Using univariable logistic regression, farm level and other related extrinsic factors were evaluated for their effect on the occurrence of subclinical mastitis as indicated on Table 5. Among all, only the presence of history of mastitis in the herd and/or cow showed a statistically significant association with the prevalence of subclinical mastitis (*p* = 0.020). Other factors such as district, kebele, type of floor of the barn, frequency of manure cleaning, practice of hand washing, and udder washing practice were found to be statistically insignificant.

**TABLE 5:** Farm‐level factors associated with prevalence of SCM in Tocha and Essera districts of Dawuro Zone (January–November 2024).

Variables	Level/category	Number examined	Prevalence		*p* value	COR (95% CI)
			Number positive	%		
District	Tocha	179	56	31.28		Ref.
	Essera	183	51	27.87	0.477	0.849 (0.54–1.333)

Kebele	Medhanealem	61	18	29.5	0.692	Ref.
	Edget	60	21	35	0.325	0.661 (0.29–1.507)
	Botori	58	17	29.31	0.108	0.514 (0.228–1.537)
	Bale	60	20	33.33	0.342	0.664 (0.289–1.537)
	Kambo	63	18	28.57	0.155	0.553 (0.245–1.251)
	Sengeti	60	13	21.67	0.379	691 (0.304–1.573)

Floor type of the barn	Wooden	205	58	28.29	0.667	Ref.
	Soil/muddy	144	46	31.94	0.685	0.760 (0.202–2.862)
	Concrete	13	3	23.07	0.512	0.639 (0.168–2.433)

Frequency of manure cleaning	Daily	130	41	31.53	0.598	Ref.
	Weekly	150	40	26.66	0.979	1.008 (0.556–1.826)
	Any time when dirty	82	26	31.7	0.416	1.277 (0.708–2.302)

Practice of hand washing	Before milking	40	13	32.5	0.914	0.921 (0.437–1.941)
	After milking	154	43	27.92	0.828	0.921 (0.437–1.941)
	Both before and after milking	15	4	26.67	0.59	1.145 (0.700–1.872)
	No washing at all	153	47	30.71	0.745	1.219 (0.369–4.027)

Udder washing	Washing	112	30	26.78	0.402	Ref.
	Washing and drying	35	8	22.85	0.322	1.292 (0.778–2.144)
	No washing	215	69	32.09	0.276	1.595 (0.689–3.692)

History of mastitis	Yes	136	50	36.76	0.020	0.580 (0.366–0.919)
	No	226	57	25.22		Ref.

#### 3.2.3. Multivariate Logistic Regression Analysis

The factors such as breed, lactation stage, and previous history of mastitis showed a trend toward significance with the occurrence of subclinical mastitis using univariable logistic regression. Other factors such as age, parity, depth of the udder, and tick infestation have *p* value less than 0.25. Before including these seven variables into multivariate logistic regression analysis, colinearity between these variables was tested by collinearity statistics such as tolerance and variance inflation factor. Accordingly, tolerance was greater than 0.1 and the variance inflation factor was less than 10, indicating the absence of collinearity between variables. Therefore, factors such as age, breed, parity, lactation period, depth of the udder, tick infestation, and previous history of mastitis in the herd and/cow were selected for further multivariable logistic regression analysis.

Among factors included in multivariable logistic regression analysis, breed (*p* = 0.007), lactation stage (*p* ≤ 0.001 for mid‐lactation, *p* = 0.030 for late lactation), and previous history of mastitis were found to had a significant relationship with prevalence of subclinical mastitis in the study area. Cross breed cows were 1.985 times more affected than indigenous or local breed cows (95% CI = 1.205–3.269). Hence, the type of cow breed was statistically associated with subclinical mastitis in the study area (*p* = 0.007). On the other hand, cows on mid‐lactation (28.22%) and late lactation stages (41.44%) were highly affected by subclinical mastitis than cows at the early stage of lactation (20.47%). The odds of getting subclinical mastitis were 1.804 times higher in herds with previous history of mastitis than herds with no history of mastitis (95% CI = 1.804–1.106) (Table 6).

**TABLE 6:** Multivariant logistic regression analysis of factors for SCM in Tocha and Essera districts of Dawuro Zone (January–November 2024).

Variables	Level/category	Number examined	Prevalence		*p* value	Adjusted OR (95% CI)
			Positive	%		
Breed	Local	190	44	23.16		Ref.
	Cross breed	172	63	36.63	0.007^∗^	1.985 (1.205–3.269)

Age	Young	111	25	22.52		Ref.
	Adult	150	48	32	0.388	1.754 (0.490–2.274)
	Old	101	34	33.66	0.845	1.092 (0.450–2.650)

Parity	Few	137	33	24.08		Ref.
	Moderate	152	49	32.24	0.801	1.176 (0.333–4.146)
	Many	73	25	34.25	0.808	1.120 (0.449–2.793)

Lactation period	Early	127	26	20.47		Ref.
	Medium	124	35	28.22	< 0.001^∗^	3.021 (1.652–5.523)
	Late	111	46	41.44	0.030^∗^	1.872 (1.063–3.296)

Depth of udder	Normal	266	74	27.82		Ref.
	Pendulous	96	33	34.37	0.901	0.960 (0.508–1.814)

Tick on udder	No	217	59	27.2		Ref.
	Yes	145	48	33.1	0.228	0.740 (0.453–1.207)

History of mastitis	Yes	136	50	36.76	0.018^∗^	1.804 (1.106–2.940)
	No	226	57	25.22		Ref.

^∗^significance.

### 3.3. Bacterial Isolation and Identification

Bacteriological samples were only taken from cows with subclinical mastitis. Accordingly, a total of 107 CMT‐positive milk samples were collected from the six kebeles of the two districts and subjected to bacteriological examination. About 103 (96.26%) of the CMT‐positive samples were found to be culture positive, the remaining 4 (3.74%) samples being culture negative. *S. aureus* was isolated from 38 samples. Therefore, the overall prevalence of *S. aureus* from subclinical mastitis was 35.5% (38/107) (Table 7).

**TABLE 7:** Prevalence of *S. aureus* in Tocha and Essera districts of Dawuro Zone (January–November 2024).

Name of district	Name of kebele	Total cultured samples	Positive for *S. aureus*	Prevalence (%)
Essera	Bale	20	9	45
	Kambo	18	5	27.77
	Sengeti	13	3	23.07
	Subtotal	*51*	*17*	33.33

Tocha	Medhanealem	18	7	38.89
	Edget	21	8	38.1
	Botori	17	6	35.29
	Subtotal	*56*	*21*	37.5

Total		107	38	35.51

### 3.4. Antimicrobial Sensitivity Testing of *S. aureus* Isolates

A total of 38 isolates of *S. aureus* indicated in Table 8 were tested against 10 antimicrobial agents according to CLSI guidelines. The test results of the 38 *S. aureus* isolates showed that 92.1%, 89.47%, 86.84%, 81.57%, and 78.95% of isolates were susceptible to cotrimoxazole (trimethoprim + sulfamethoxazole), kanamycin, streptomycin, sulfisoxazole, and erythromycin, respectively. In addition to this, 36.84% (14/38) and 28.95% (11/38) of *S. aureus* were susceptible to oxytetracycline and tetracycline, respectively. In contrast, the highest resistance development was observed for penicillin G (100%), followed by ampicillin 97.37% (37/38), oxacillin 89.47% (34/38), tetracycline 68.42% (26/38), and oxytetracycline 52.63% (20/38). In this study, 18.42%, 15.79%, 10.53%, 10.53%, 7.89%, and 2.63% of isolated *S. aureus* showed intermediate resistance to sulfisoxazole, erythromycin, kanamycin, streptomycin, oxytetracycline, cotrimoxazole (trimethoprim + sulfamethoxazole), and tetracycline, respectively.

**TABLE 8:** Antimicrobial susceptibility testing of isolated *S. aureus* in Tocha and Essera districts of Dawuro Zone (January–November 2024).

Class of antibiotics	Antimicrobials	Susceptible		Intermediate		Resistant	
		*n*	%	*n*	%	*n*	%
Tetracyclines	Tetracycline	11	28.95	1	2.63	26	68.42
	Oxytetracycline	14	36.84	4	10.52	20	52.63

Sulfonamides	Sulfisoxazole	31	81.57	7	18.42	0	0
	Cotrimoxazole	35	92.1	3	7.89	0	0

Macrolides	Erythromycin	30	78.95	6	15.79	2	5.26

Penicillins	Penicillin G	0	0	0	0	38	100
	Ampicillin	1	2.63	0	0	37	97.37
	Oxacillin	4	10.52	0	0	34	89.47

Aminoglycosides	Kanamycin BS	34	89.47	4	10.53	0	0
	Streptomycin NS	33	86.84	4	10.53	1	2.63

In the present study, multidrug‐resistant isolates of *S. aureus* were also identified. A total of two, out of 38 isolates (5.26%), were multidrug resistant; they were resistant to antibiotics belonging to three (*n* = 1) and four classes (*n* = 1) of drugs. Majority of the isolates were highly resistant to almost all of β‐lactam (penicillins) class of drugs. For instance, 89.47% (34/38) of the isolates were resistant to three antibiotics from penicillins (β‐lactam) class of antibiotics. About 68.42% and 50% of isolates were resistant to four and five antibiotics from the two classes of antibiotics (penicillins and tetracyclines), respectively (Table 9).

**TABLE 9:** Multidrug resistance *S. aureus* in Tocha and Essera districts of Dawuro Zone (January–November 2024).

Class of antimicrobials	Antimicrobials	No. of resistant isolates	Percent
Penicillins (B‐lactam)	P‐AMP	37	97.37
	P‐AMP‐OX	34	89.47

Penicillins and tetracyclines	TE‐P‐AMP‐OX	26	68.42
	TE‐OT‐P‐AMP‐OX	19	50

Penicillins, macrolides, and aminoglycosides	E‐P‐AMP‐ST	1	2.6

Penicillins, tetracycline, macrolides and aminoglycosides	TE‐OT‐E‐P‐AMP‐OX	1	2.6

*Note:* P—penicillin G, AMP—ampicillin, OX—oxacillin, TE—tetracycline, OX—oxytetracycline, E—erythromycin, ST—streptomycin.

## 4. Discussion

In the present study, out of 384 cows examined, 129 (33.53%) were infected by bovine mastitis, out of which, 107 (27.86%) were infected by subclinical mastitis and 22 (5.73%) of which were found to be clinically infected. The overall prevalence (33.53%) of mastitis in this study was comparable with the finding of Yimana and Tesfaye [36] in Adama (31.5%), and it was higher than 29.09% of Shire dairy farms in Tigray region [12], 30.21% of Bench Maji [37], and 30.5% by Ref. [13]. However, it is lower than 39.67% in West Wollega [15], 50.8% in and around Hawassa [16], 54.9% in Silte [17], 72.8% in and around Modjo [19], and 73% in Borana district of South Wollo [18]. The differences in the prevalence of bovine mastitis between the present finding and the previous studies could be due to the fact that mastitis is a complex disease associated with different risk factors, the difference in the farm management systems, improper animal health and hygienic management, agro‐climatic changes, and differences in study methods and instruments used by the investigators [38].

The present study revealed 5.73% prevalence of clinical mastitis. This was comparable with 5% in Sululta and Holeta [39], 6.1% in Southern Ethiopia [40], and 6.8% in Kenya [41], and it was higher than the prevalence (2.9%) recorded in Silte [17]. However, the prevalence reported in the present findings was lower than that reported in the previous studies from different parts of Ethiopia [15, 19, 21, 42, 43].

The prevalence of subclinical mastitis (29.55%) observed in this study was much higher than the occurrence of clinical cases. This finding was comparable with previous studies reported by Melesse and Minyahil [44] in the mid‐rift valley of Ethiopia (28.2%), Tezera and Aman Ali [14] in Asosa town of Benshangul Gumuz (28.34%), Michira et al. [45] in Kajiado Central subcounty of Kenya (31.5%), Ejeta and Pal [42] in Ambo (32.6%), and Endeshaw et al. [46] in Northwest Ethiopia (32.7%). Lower prevalence of subclinical bovine mastitis was recorded in this study than other studies in southern Tigray Baraki et al. [47], in North Upper Egypt Abed et al. [48], in Kajiado Central subcounty of Kenya [45], in Central Ethiopia [49], in Eastern Ethiopia Ahmed et al. [50], in Rwanda [51], in Sululta and Holeta town [39], and in Tanzania [52] who reported prevalence of 44.52%, 46%, 49%, 53%, 54.3%, 62.0%, 66.7%, and 70.8%, respectively. However, this finding was higher than previous studies in different regions of Ethiopia, for instance, 15.2% in Gamo zone [53], 21.8% in West Gojam [54], and 22.7% in West Wollega [15]. The current study including the report of researchers mentioned in this section indicates higher prevalence of subclinical mastitis over clinical mastitis. The high prevalence of subclinical mastitis has a negative impact on dairy cow health and productivity as well as health of the public. It serves as a source of zoonosis. The high occurrence of subclinical mastitis over clinical form could be due to the fact that clinical mastitis can easily be diagnosed and treated while subclinical form had no visible symptoms, which was therefore difficult to be identified by the farmers and progresses to be a source of infection in the cattle farm [38].

Multivariate logistic regression analysis of factors associated with occurrence of subclinical mastitis in the present study revealed that breed, lactation stage, and previous history of mastitis had significant association with the occurrence of subclinical mastitis. The prevalence of subclinical mastitis was significantly higher in cross breed dairy cows than in indigenous breed cows (*p* = 0.007, AOR = 1.985, CI = 1.205–3.269). This finding agrees with the study in different parts of Ethiopia [14, 21, 50, 54, 55] and in Kenya [45]). This finding indicates that zebu breeds of cows in the study area were found to be resistant to subclinical mastitis as compared to cross breeds. This is due to the fact that local zebu breeds have higher natural immunity for disease and adaptability to unfavorable conditions than exotic breeds [38].

Subclinical mastitis was higher in mid‐lactation (*p* = 0.000) and late lactation (*p* = 0.03). The likelihood of getting subclinical mastitis was 3.021 times higher in mid‐lactation than in the early stage of lactation. The disease was also 1.872 times higher on cows at the late stage of lactation than on cows in the early stage of lactation. This agrees with other previous studies in west Shewa [13], in Eastern Ethiopia [50], in Northwest Ethiopia [46], in Wolmera district of Oromia region of Ethiopia [55], in Kembata South Ethiopia [28], and in Rwanda [51]. On contrary, other studies in some parts of Ethiopia ([14, 54, 56]) and in other countries [57] revealed high occurrence of subclinical mastitis in the early stage of lactation than mid‐ and late stage of lactation. The current result of increasing prevalence as the lactation period increases might be related to the prolonged exposure time of milking and to pathogens from dirty ground with increasing ease of penetration of the teat duct by these pathogens and also previously accumulated pathogens due to insufficient diagnosis and treatment. Chronic mastitis, most often subclinical, is more frequent later during lactation [38].

The prevalence of subclinical mastitis was higher in cows/herds with previous history of mastitis (*p* = 0.018, AOR = 1.804, CI = 1.106–2.940). From a total of 362 lactating cows examined, 135 cows had a previous history of mastitis, of which 36.29% were mastitis positive. This significant association was in line with the study done in other parts of Ethiopia [13, 14, 58] and [57] Nepal. This could be because of the lack of screening tests and treatment for subclinical mastitis, as well as insufficient follow‐up for clinical and chronic mastitis in the study area, and could lead to the prolonged existence of organisms in mammary tissue.

Out of 107 CMT‐positive samples cultured, 103 (96.2%) of samples were found to be culture positive for single or more bacterial species and the remaining 3.74% (4/107) were culture negative. This result was lower than that reported in the study by Berhe [59] who reported 6.56% negative culture. The failure to isolate bacteria from the CMT‐positive milk samples could be due to the spontaneous elimination of the infection, small amount of pathogen in milk, intermittent shedding and intracellular location of pathogens, and presence of inhibitory substance in milk [31] or might be due to the continued infiltration of leukocytes until complete healing [60].

In the present study, the overall prevalence of *S. aureus* in subclinical mastitis was recorded to be 35.51% (38/107). This finding was comparable with the findings of Bihon et al. [56] who reported 34% of prevalence and it was lower than other previous studies ([16, 43, 48, 49, 59, 61] who reported 42.43%, 42.1%, 44.12%, 44.9%, 47.8%, and 68%, respectively). Poor hygienic management and poor treatment of clinical mastitis with antibiotics without selection (without selection for susceptibility) contributed to the increased occurrence of *S. aureus* in the current study area. Moreover, it is known that Staphylococcus species are adapted to survive in the udder and usually establish chronic subclinical infection of long duration, from which it will shed through milk, serving as a source of infection for other healthy cows and also transmitted during the milking process [38].

Antimicrobial susceptibility testing of isolated *S. aureus* in the current study revealed that isolates of *S. aureus* were susceptible to cotrimoxazole(trimethoprim + sulfamethoxazole) (92.1%), kanamycin (89.47%), streptomycin (86.84%), sulfisoxazole (81.57%), and erythromycin (78.95%). Both streptomycin and kanamycin are aminoglycosides used to treat animal diseases including mastitis. Streptomycin is available as combination with penicillin G termed as Penstrep, which was one of the preferable injectable drugs by animal health workers used to treat mastitis in the study area. Kanamycin is available as intramammary infusion used to treat mastitis. A comparable susceptibility to cotrimoxazole (trimethoprim + sulfamethoxazole) was reported by Refs. [43, 61]. In contrast to current finding, 66.7% resistance to cotrimoxazole (trimethoprim + sulfamethoxazole) was reported by Ref. [58] in Meta district of Eastern Ethiopia. The current finding on susceptibility to Kanamycin was in line with Refs. [58, 62]. A study in Bedele and Nekemte towns of Oromia region [18] showed a similar level of susceptibility of *S. aureus* to erythromycin (77.8%) and streptomycin (88.9%). However, the report of Ref. [63] in Shashemene showed that 54.5% and 50% of isolates were resistant to erythromycin and streptomycin, respectively. Moreover, another study in Central Ethiopia [62] and from Kenya [45] showed resistance of *S. aureus* against erythromycin and streptomycin.

On the other hand, the current study revealed that the isolated *S. aureus* had developed resistance to common veterinary drugs, which are currently used for the treatment of mastitis and other animal diseases. The isolates had developed complete resistance (100%) to penicillin G. This was similar to the study by Refs. [28, 43, 58, 62] in different parts of Ethiopia. *S. aureus* was also developed very high resistance (97.37%) to another type of penicillin (B‐lactam class), a broad‐spectrum betalactamase‐sensitive penicillin called ampicillin. A comparable resistance against ampicillin was recorded by Ref. [22] in Ambo and Bako town of Oromia region of Ethiopia (98.48%) [63], in Shashemene Town (90%) and [48] in upper Egypt (97%). Oxacillin is one of penicillinase stable penicillin, which have been used for the therapy of penicillin G resistance staphylococcal infections. However, the current study revealed 89.47% resistance against oxacillin. This was the result of inappropriate use of these medications by animal health workers and others in the study, which contributed to the emergence of resistance, since penicillins have been used for long periods as a choice of drug for the treatment of mastitis. It is a documented fact that some bacteria like *S. aureus* developed resistance to penicillin G and ampicillin by the mechanism of antibiotic inactivation, where *S. aureus* produce enzymes called β‐lactamases, which hydrolyze the β‐lactam ring of these penicillins causing the inactivation of drugs before they reach their target within the bacteria [64]. In addition, the development of resistance against penicillin group of drugs might be due to the ability of *S. aureus* to modify the penicillin target by variously altering the penicillin‐binding protein (PBP), which is the target of β‐lactam [65].

Tetracyclines are a broad‐spectrum antibiotic, which are the most commonly used drugs by animal health workers nowadays everywhere in Ethiopia including current study area. However, the current isolates of *S. aureus* were found to be resistance to tetracycline (68.42%) and oxytetracycline (52.63%). The result was in line with Refs. [58, 59] who reported that 69.2% and 50% of isolates developed resistant to tetracycline, respectively. However, the lower level of resistance was recorded in the present study as compared to the finding of Feyissa et al. [43] (100% resistance to tetracycline and oxytetracycline) and in Ambo and Bako towns of Oromia region [22] who reported 88.3% resistance against tetracycline.

Multidrug resistance (MDR) is acquired nonsusceptibility to at least one agent in three or more antimicrobial categories [66]. In the present study, about 5.26% (*n* = 2) of *S. aureus* isolates got MDR: One isolate (2.6%) was resistant to four antibiotics from three classes of antibiotics and another one isolate (2.6%) was resistant to six antibiotics from four classes of antibiotics. A comparable prevalence of MDR was reported by Kalayu et al. [67] from Mekele, Northern Ethiopia. The prevalence of the present study was highly lower than that reported in the study by Holeta [68], by Shinshicho town of Kembata Tembaro [28], and by Bako town [22] who reported 65%, 53.2%, and 43.94% of the isolates got MDR, respectively. The emergence of MDR poses a risk to public health because treatment for food‐borne outbreaks may be challenging and the group of MDR *S. aureus* in the food supply acts as a reservoir for communicable resistant genes [69].

The current study revealed *S. aureus* has got alarming levels of resistance to currently available and commonly used antimicrobials including penicillin G, amoxicillin, tetracycline, and oxytetracycline in the study area. This could be caused by the erratic and extensive use of antibiotics without prior antimicrobial susceptibility testing. Since, antibiotic‐resistant *S. aureus* isolates pose a challenge to both animal and public health, it needs special attention on appropriate use of veterinary drugs.

## 5. Conclusion

The present study showed high prevalence of mastitis in dairy herds, indicating that mastitis was a one among disease of dairy cows, which undoubtedly decrease productivity. In particular, the subclinical mastitis was highly prevalent than clinical form showing the lack of concern and unawareness on subclinical mastitis. Breed of the cow, lactation period, and previous history of mastitis in herd and/cows showed a significant association with subclinical mastitis. Bacteriological characterization showed that *S. aureus* was widely prevalent in raw milk of cows with subclinical mastitis in Tocha and Essera districts of Dawuro Zone. The isolates of *S. aureus* were sensitive to Co‐Trimoxazole (trimethoprim + sulfamethoxazole), kanamycin, streptomycin, sulfisoxazole, and erythromycin. However, the isolates have developed the highest resistance against penicillin G followed by ampicillin, oxacillin, tetracycline, and oxytetracycline. Moreover, some isolates of *S. aureus* showed multiple drug resistance against commonly used antibiotics. The high prevalence of drug‐resistant and multiple drug–resistant *S. aureus* in subclinical mastitis poses great challenge to public health, animal health, and welfare and economy. Therefore, regular screening for subclinical mastitis using CMT and proper treatment of positive cases in the herd; conducting an in vitro antibiotic susceptibility test prior to the use of antimicrobial agents; and creating awareness for the community to practice good animal health management and to avoid consuming raw milk or improperly prepared milk and milk products to avoid the transmission of pathogenic and drug resistance *S. aureus* were recommended to minimize the impact of the disease. Moreover, a multicentered research should be conducted on susceptibility of antimicrobials to inform formal policy updates on the use of veterinary drugs in the study area.

## Author Contributions

Ephrem Gebrehiwot and Mekonnen Addis conceived the study idea. Ephrem Gebrehiwot, Tamrat Haile, and Tsegaye Teklemariam were involved in designing the research and data collection. Ephrem Gebrehiwot, Tamrat Haile, and Tsegaye Teklemariam performed laboratory investigation. Ephrem Gebrehiwot, Mekonnen Addis, and Mukarim Abdurahman were involved in data analysis and manuscript draft preparation. Ephrem Gebrehiwot, Mekonnen Addis, Mukarim Abdurahman, Tamrat Haile, and Tsegaye Teklemariam reviewed the manuscript.

## Funding

No funding was received for this manuscript.

## Disclosure

All authors read and approved the final version of the manuscript.

## Ethics Statement

The current research study was approved by the Jimma University College of Agriculture and Veterinary Medicine institutional ethical review board by approval letter, Ref. No. VetMed/31/2026. Survey protocols and animal handling methods were done according to the required guideline, which was approved by the ethical review board. The purpose of the study was explained for the participants before the commencement of the study.

## Conflicts of Interest

The authors declare no conflicts of interest.

## Supporting Information

Additional supporting information can be found online in the Supporting Information section.

## Supporting information


**Supporting Information** Supporting File 1: Questionnaire used for data collection in the study area.

## Data Availability

All data supporting these research findings are included within the manuscript. The databases (without personally identifiable information) are available from the corresponding author upon request.
