# Supplementary material for: Prevalence of Mastitis, Bacteriological Identification, and Antibiogram of Staphylococcus aureus From Subclinically Infected Lactating Cows in Tocha and Essera Districts, Dawuro Zone, Southwest Ethiopia
Source: Vet Med Int. 2026 Oct 15;2026:3884904. doi: 10.1155/vmi/3884904 (PMC13673006; doi:10.1155/vmi/3884904)
Supplement: Supplementary file 1 [file VMI-2026-3884904-s001.docx]

**Questionnaire Format**

Date of sample Collection______________

1. General information
   1.1. Owners Name:	_
   1.2. Address:-- District________Kebele_______ Phone Number	____________
2. Herd/farm and Housing information
   2.1. Herd _____________ farm____________
   2.2. Herd size 1) Small (<5)	2) Medium (6-10)	3) Large (>11)
   2.3. Housing system	1) Separate Pen	2) Group (shared) barn
   2.4. Is there bedding material? 1) Yes	2) No
   2.5. Type of floor in cow house/farm 1) wooden 2) muddy/soil 3) concrete
   2.6. How often do you clean manure/bedding? 1) Daily	2) weekly	3) any time if dirty	4) Others (specify)
   2.7. General hygiene of the farm observed	1) Good	2) Poor
3. History of each sampled cow
   3.1. ID No________
   3.2. Breed 1) Local/zebu/	2) Cross breed
   3.3. Age	1) 3-5 (Young)	2) 6-8 (Adults)	3) ≥9 (Old)
   3.4. Body condition	1) Good	2) medium	3) poor
   3.5. Parity: 1) few (1-2 calves) 2) moderate (3-5 calves) 3) Many (≥6)
   3.6. Lactation stage of the cow? 1) Early (1-3)	2) Medium (4-6)	3) Late (>=7)
   3.7. How many litres of milk do you get per day?	1) Low (1–5L)	2) medium (6–10L) 3) high (≥11L).
4. Physical inspection of the udder, teat and milk
   4.1. What is udder & leg hygiene of Cow?	1) Slightly dirty	2) Moderately dirty	3) Very dirty
   4.2. What is the depth of Udder?	1) Normal	2) Pendulous
   4.3. Is there lesion on the udder/ teat?	1) Yes,	2) No, it is absent
   4.4. Is there any inflammatory sign (Heat, pain, swelling) on the udder and teat? 1) Yes 2) No
   4.5. Is there any blindness of teat canal? 1) Yes 2) No
   4.6. Is there any tick infestation on teat? 1) Yes, it is present 2) No it is absent
   4.7. What is gross milk quality? 1) Normal 2) watery 3) clots/flakes 4) blood tinged/pus
5. Status of hygienic milking practice
   5.1. How do you milk your cow?1) Milking with hand2) milking with machine
   5.2. When do you wash your hand for milking? 1) Before milking only 2) After milking only 3) Before and after milking 4) between milking processes 5) Not at all
   5.3. When do you wash the udder? 1) Before milking only 2) After milking only

3) Both before and after milking 4) No Washing At all

5.4. What type of udder washing do you follow? 1) Washing &drying 2) washing only
5.5. Do you use drying towel? 1) Yes I do, I use separate Towel for each cow 2) Yes I do, I Use common towel for all cow 3) No, I don’t use any drying towel
5.6. Do you use post milking dipping by alcohol? 1) Yes, I do	2) No, I don’t

6. Mastitis Situations
   6.1. Do you know (have you heard) about clinical mastitis cases? 1) Yes	2) No
   6.2. Do you know (have you heard) about subclinical mastitis cases? 1) Yes	2) No
   6.3. Is there previous exposure to mastitis problem in the farm? 1) Yes	2) No
   6.4. When do you milk the cows with mastitis?	1) First	2) Last	3) Any time
   6.5. Do you treat mastitis case as they occur? 1) Yes	2) No
   6.6. Do you know the name of drugs used for mastitis treatments? 1) Yes	2) No
   6.7. Have you ever practiced Culling of chronically infected cows? 1) Yes	2) No
